# From malaria control to elimination in South Africa: The researchers’ perspectives

**DOI:** 10.4102/phcfm.v8i1.1078

**Published:** 2016-07-29

**Authors:** Khumbulani W. Hlongwana, Joyce Tsoka-Gwegweni

**Affiliations:** 1 School of Nursing & Public Health, College of Health Sciences, University of KwaZulu-Natal, South Africa

## Abstract

**Background:**

Global decline in malaria episodes over the past decade gave rise to a debate to target malaria elimination in eligible countries. However, investigation regarding researchers’ perspectives on barriers and facilitating factors to effective implementation of a malaria elimination policy in South Africa (SA) is lacking.

**Aim:**

The aim of this study was to investigate the malaria researchers’ knowledge, understandings, perceived roles, and their perspectives on the factors influencing implementation of a malaria elimination policy in SA.

**Setting:**

Participants were drawn from the researchers who fulfilled the eligibility criteria as per the protocol, and the criteria were not setting-specific.

**Methods:**

The study was a descriptive cross-sectional survey conducted through an emailed self-administered semi-structured questionnaire amongst malaria researchers who met the set selection criteria and signed informed consent.

**Results:**

Most (92.3%) participants knew about SA’s malaria elimination policy, but only 45.8% had fully read it. The majority held a strong view that SA’s 2018 elimination target was not realistic, citing that the policy had neither been properly adapted to the country’s operational setting nor sufficiently disseminated to all relevant healthcare workers. Key concerns raised were lack of new tools, resources, and capacity to fight malaria; poor cross-border collaborations; overreliance on partners to implement; poor community involvement; and poor surveillance.

**Conclusion:**

Malaria elimination is a noble idea, with sharp divisions. However, there is a general agreement that elimination requires: (a) strong cross-border initiatives; (b) deployment of adequate resources; (c) sustainable multistakeholder support and collaboration; (d) good surveillance systems; and (e) availability and use of all effective intervention tools.

## Introduction

Documented evidence demonstrates that malaria incidences continue to decline, globally.^1^ In 2013, 198 million malaria cases and 584 000 deaths occurred, globally, translating into a decrease in mortality and case incidence by 47% and 30%, respectively, over 13 years (2000–2013).^2^ Sub-Saharan Africa continues to bear the highest (90%) disease burden worldwide.^2^ However, the last decade in South Africa (SA) has been hailed for substantially reducing the burden of malaria in the country.^3,4^ These achievements were also noted elsewhere in the world, giving rise to a new debate to target malaria elimination in countries where malaria had been substantially reduced.^5,6^ South Africa, Swaziland, Namibia, and Botswana were amongst the first countries in Southern Africa to be declared by the World Health Organization (WHO) as ready to eliminate malaria.^3^

Since 2007, malaria elimination has become a topical issue in the international malaria community, which includes funding agencies, malariologists, and interventionists.^1,7^ However, the setbacks of the Global Malaria Eradication Programme (GMEP) of 1955–1969^8^ would inevitably have implications on how a malaria elimination programme is perceived and/or implemented today. Despite partial successes, one of the serious shortcomings of the GMEP of 1955–1969 was the fact that the entire programme was premised on the assumptions that all the necessary knowledge was available, and any further research was superfluous.^8^ At the time the programme was abandoned, it was realised that most experienced malariologists had migrated out of the system because of a shift in priority focus.^8^

South Africa was already at the pre-elimination phase in 2010, with an incidence of 1.14 cases per 1000 population at risk.^3,4^ In 2013, the at-risk population from the three malaria endemic provinces (KwaZulu-Natal, Limpopo, and Mpumalanga) was about 5 277 613 people.^2^ *Plasmodium falciparum* remains the most prevalent parasite, accounting for approximately 95% of all malaria cases in SA.^9^ The main malaria vector is *Anopheles* *arabiensis*.^10^

Upon a closer look, it becomes apparent that the three malaria endemic provinces in SA and their respective districts are at different phases of the malaria elimination continuum. For example, all 11 districts in KwaZulu-Natal have a malarial incidence of less than one case per thousand population at risk, thus qualifying for transition to elimination, whereas in Mpumalanga and Limpopo, some of the districts or parts thereof are still at the control phase.^3^ These differences can be attributed to a number of factors, including (a) differences in malarial parasite susceptibility to the drugs of choice; (b) variability in climatic conditions; (c) socio-economic conditions’ impacts on dwelling types; (d) population migratory patterns; and (e) evolving complexities concerning malaria epidemiology in these areas.^11^

Although malaria elimination remains a subject of intense local and international debate, to date there is little or no evidence to suggest that studies concerning the barriers and facilitating factors to effective implementation of a malaria elimination policy in SA and elsewhere in the world have been conducted, at least from the perspectives of the malaria researchers. The aim of this study was to investigate the malaria researchers’ knowledge and understanding of the malaria elimination policy and seek their perspectives on the factors that influence the implementation of the policy in SA. Their perceived roles in the implementation of a malaria elimination programme were also investigated.

## Research method and design

### Study design

The study was a descriptive cross-sectional survey conducted through an emailed self-administered semi-structured questionnaire amongst malaria researchers who met the set selection criteria and signed informed consent.

### Setting

This study did not require participants to be based in any specific geographical or institutional settings, as long as they fulfilled the eligibility criteria as set out in the subsequent section. Although most research participants were based in SA, a few resided in other countries, such as, the United Kingdom, Canada, and Zimbabwe.

### Study population and sampling strategy

The study population constituted all researchers who published malaria-related research articles based in SA during the period 01 January 2008 to 31 December 2013, either as first, last, or corresponding authors. These researchers were identified through electronic search engines PubMed, Google Scholar, and Science Direct. These timelines were motivated by the fact that the world leaders made their first call for malaria elimination in 2007^12^ and by 2008 researchers who were involved in malaria research were likely to have been aware of the imminent malaria elimination agenda.

**FIGURE 1:**
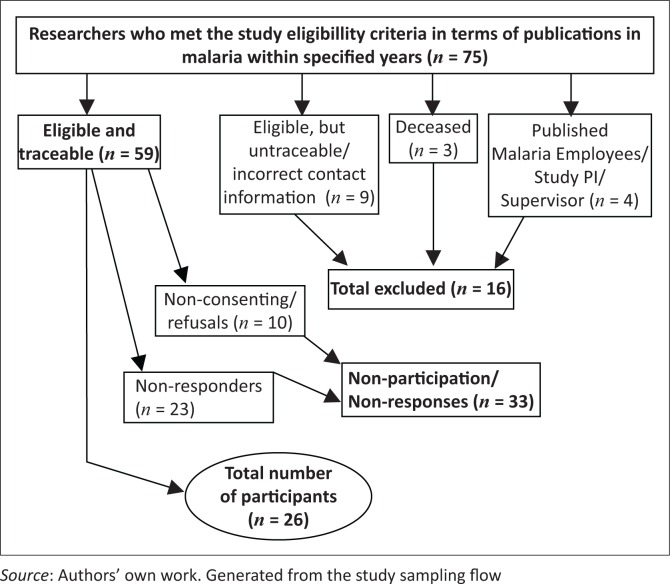
Participant selection flow chart.

Based on the order of authorship of published studies, 75 researchers met the set criteria. Authors of case study articles whereby SA was either a case study, or directly compared with other countries, were considered for inclusion. Authors of articles or reviews focussing on malaria at a regional level, for example, Southern Africa, Sub-Saharan Africa, or Africa, were excluded on the basis that SA was unlikely to be a focal point in such articles. All non-consenting eligible malaria researchers were excluded from the study, resulting in a sample size of 26 participants, which was 44.1% of all eligible and traceable researchers (Figure 1).

### Inclusion and exclusion criteria

All researchers who published malaria-related research in SA during the period spanning from 2008 to 2013 were eligible for inclusion into the study. In the case of co-authored papers, only the first, last, and or corresponding authors were included in the study as they were likely to have had considerable contribution into such studies and a better insight into malaria situation in SA. Malaria control employees who met the publication criteria were not included in the study to avoid conflicted roles, because their core functions lay in the malaria control/elimination activities.

Eligible researchers were not excluded on the basis of the country of residence. Authors of case study articles whereby SA was either a case study, or directly compared with other countries, were included. Authors of articles or reviews focussing on malaria at a regional level, for example, Southern Africa, Sub-Saharan Africa, or Africa, were excluded, because SA would not have been a key focus in such articles. The academic supervisor for this study was excluded to avoid conflict of interest. All authors working for the Department of Health or any government institution other than the malaria programme were included, as long as they satisfied the criteria. All non-consenting eligible malaria researchers were excluded from the study.

### Data collection

Data were collected through the use of semi-structured questionnaires, which, together with a participant information sheet and consent form, were emailed to all identified eligible researchers for self-administration. Subsequently, three more follow-up reminders were made at 3 to 4 weeks intervals. In special circumstances, whereby potential participants had shown interest in participating in the study and requested to be reminded, the number of follow-up emails exceeded the normal three reminders. However, in certain circumstances additional reminders did not produce positive outcomes, as anticipated. One potential participant requested further information about the study, after which she comfortably completed the questionnaire.

The questionnaire was adapted in the tools of published studies,^13^ and presented in various fora to improve validity. These fora included presentations at the departmental (Discipline of Public Health Medicine) journal clubs and PhD cohort sessions, whereby all PhD students presented their work to a panel of supervisors and peer students, on a quarterly basis. Both peer students and different PhD supervisors provided feedback and suggestions for improvements.

### Data analysis

Data were entered into the Epi Info Database, checked for errors and duplications, and analysed through the descriptive statistics using SPSS version 23 software.

## Ethical considerations

This paper is part of a PhD study, which obtained the full ethics approval (REF: BE240/14) from the University of KwaZulu-Natal Biomedical Research Ethics Committee (BREC). All potential research participants received participant information sheets explaining the study prior to signing the informed consent form.

## Results

Of the 75 identified researchers, 16 (21.3%) were excluded because: (a) they were untraceable or had incorrect contact information; (b) authors were employees of malaria control programmes; and (c) authors were principal investigators or supervisors for the current study. Of the remaining 59 eligible and supposedly traceable researchers, 10 (16.9%) were refusals and 23 (39.0%) were non-responders. Some non-responders may actually belong to untraceable, because all emails that did not bounce were assumed to be valid and functional. Refusals had the following as reasons for non-participation:

- The scope of the study was irrelevant and fell outside their research interests.
- They felt outdated about malaria developments in SA.
- They considered their exposure to malaria research in SA to be limited.
- They felt uncomfortable about participating in this study, as they had neither seen nor could find the final elimination document through web searches.
- They were too busy to have time to complete a survey questionnaire.

### Biographic information

The study participants consisted of 14 males and 12 females, with ages ranging from 30 to 88 years and the mean and standard deviation of 48.9 and 13.8 years, respectively. English was home language to most respondents (76.9%) and more than half (53.9%) of the participants had PhD qualification. Participants’ job designations varied widely, and so did their experiences in malaria research, which ranged from 1 to 42 years (Table 1), with a mean and standard deviation of 15.7 and 10.7, respectively. Almost half (46.2%) of the respondents were members of the South African Malaria Elimination Committee (SAMEC), which is a technical advisory group to the National Department of Health on matters relating to malaria elimination.

**TABLE 1:** Respondents’ biographical profile.

Variable	*n*	%
**Age in years (*n* = 23)**		
≤ 30	1	4.4
31 – 44	9	39.1
45 – 59	8	34.8
≥ 60	5	21.7
**Home language (*n* = 26)**		
English	20	76.9
Other	6	23.1
**Gender (*n* = 26)**		
Male	14	53.9
Female	12	46.2
**Highest level of education attained (*n* = 26)**		
Undergraduate degree/ diploma	1	3.9
MB ChB	6	23.1
Masters	5	19.2
PhD	14	53.9
**Job designation (*n* = 26)**		
Academic/Professor/Lecturer	5	19.2
Chief Specialist Researcher	3	11.5
Senior Specialist Researcher	3	11.5
Senior Researcher	2	7.7
Researcher/Research Consultant	4	15.4
Technical/Content Advisor	2	7.7
Family Physician	2	7.7
Infectious Diseases Specialist	1	3.9
Public Health Specialist	1	3.9
Other	3	11.5
**Years of experience (*n* = 26)**		
≤ 9	8	30.8
10 – 19	10	38.5
20 – 29	5	19.2
≥ 30	3	11.5
**Work province or country, if working outside South Africa (*n* = 26)**		
KwaZulu-Natal	6	23.1
Limpopo	1	3.9
Mpumalanga	2	7.7
Gauteng	8	30.8
Other provinces	2	7.7
National role	2	7.7
Regional role	2	7.7
Other countries (Canada, United Kingdom and Zimbabwe)	3	11.5
**Membership of South African Malaria Elimination Committee (*n* = 26)**		
Members	12	46.2
Non-members	14	53.9

*Source*: Authors’ own work. Generated from the study data

### Researchers’ knowledge and understanding of malaria elimination policy

Most (92.3%) participants had heard about SA’s malaria elimination policy, 75% of whom had seen the policy document, and 45.8% had fully read it. The content understanding of those who had read the policy, whether partially or in full, ranged from average (27.8%), good (55.6%), to very good (16.7%). Although the sample size was too small to generate any meaningful stratification of researchers’ policy awareness by biographic details, indications were that qualifications had very little effect on increased awareness, as did the job designation. For example, of the 26 respondents, only 2 (1 South African researcher and 1 Canadian-based professor, both with less than 9 years of malaria research experience) had not heard about the malaria elimination policy and both were PhD graduates. Of the 12 PhD graduates, 7 had reportedly seen the policy, and 3 had fully read it. Of the 6 MB ChB graduates, 5 reported having seen the policy and 3 had fully read it. On the other hand, all 5 Masters Graduates had reportedly seen and fully read the policy.

Strangely, the respondents’ years of experience in malaria research appeared to inconsistently affect their malaria elimination policy awareness levels. For example, all 10 researchers whose malaria research experiences ranged from 10 to 19 years had reportedly heard of and seen the malaria elimination policy, half of whom had fully read it, and the other half had partially read it. Even more puzzling was the fact that those who had ≥ 30 years of experience in malaria research had no better policy awareness than those who had experiences ≤ 9 years in malaria research.

Only 11.5% of the participants believed that the policy had been sufficiently disseminated to all relevant healthcare workers. More than half (53.9%) believed that the policy had not been properly adapted to suit SA’s malaria operational setting. Notably, some researchers were unable to identify the correct definition for ‘malaria elimination’ as defined by the WHO. The WHO’s definition of malaria elimination was listed in a questionnaire along with other incorrect definitions. Researchers were asked to identify and mark the correct definition and 72% were able to recognise the correct definition (Table 2). Characteristics of the respondents who could not identify the correct definition of malaria elimination varied widely, in terms of age and qualifications. Except for the two respondents, who got involved in malaria research in 1984 and 1994, respectively, the rest of the respondents who could not identify the correct definition of malaria elimination joined malaria research between 2001 and 2009. Therefore, there is no plausible explanation for their failure to identify the correct definition, except that they may not have given themselves time to read and understand such technical concepts.

**TABLE 2:** Participants’ knowledge of malaria elimination, as defined by the WHO.

Options provided in questionnaire	*n = 25*	%	Correct (✓) Incorrect (✕)
Permanent reduction of malaria transmission to zero at a global level	1	4	✕
No local malaria transmission over a period of three years within a defined geographical area	18	72	✓
Killing all malaria transmitting mosquitoes within a defined geographical area	2	8	✕
All of the above	0	0	✕
None of the above	2	8	✕
Don’t know	2	8	

*Source*: Authors’ own work. Generated from the study data

The target year (2018) set by SA to eliminate malaria was well known, but only one participant (3.9%) was optimistic about the target year, with 88.5% stating that the goal was not achievable within the set target, and 7.7% did not know whether the goal was achievable or not. Participants provided a range of reasons why they believed SA was not ready to eliminate malaria by 2018 (Table 3). Key reasons were the lack of new tools to fight malaria (23.1%), lack of involvement by all relevant role players (34.6%), and matters relating to cross-border and importation of malaria cases from the neighbouring malaria endemic countries (34.6%). Of the 46.2% participants who believed that malaria can be eliminated post 2018 target, they proposed varied timelines, namely 2020 (8.3%), 2024 (8.3%), 2025 (50%), 2030 (25%), and 2050 (8.3%).

**TABLE 3:** Participants’ reasons for believing the country will not eliminate malaria by 2018 (multiple responses were allowed).

Responses	*n = 26*	%
Malaria cases are still very high	2	7.7
Intervention tools used to control malaria have not changed	6	23.1
Malaria elimination is not supported by research evidence	4	15.4
Not all role players are involved in malaria elimination
programme	9	34.6
There is not enough budget to implement malaria elimination	2	7.7
Cross-border initiatives are lacking and borders are porous
resulting in high importation of cases	9	34.6
There is not enough skills to implement malaria elimination	2	7.7
Other	5	19.2

*Source*: Authors’ own work. Generated from the study data

### Researchers’ perceived roles in implementing malaria elimination policy in South Africa

**FIGURE 2:**
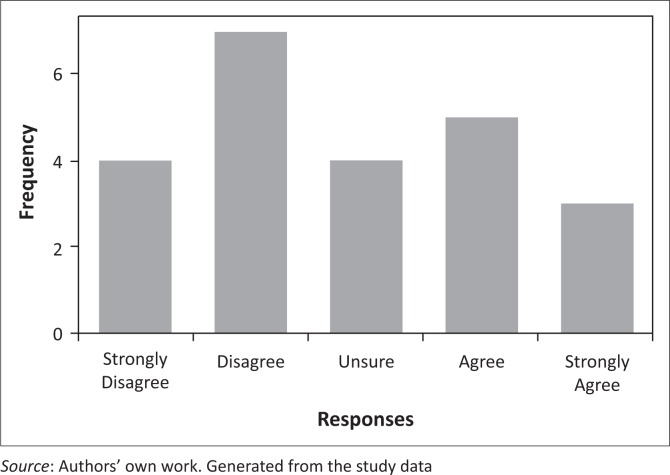
Scaled responses regarding the adequacy of malaria researchers to guide malaria elimination in SA.

**FIGURE 3:**
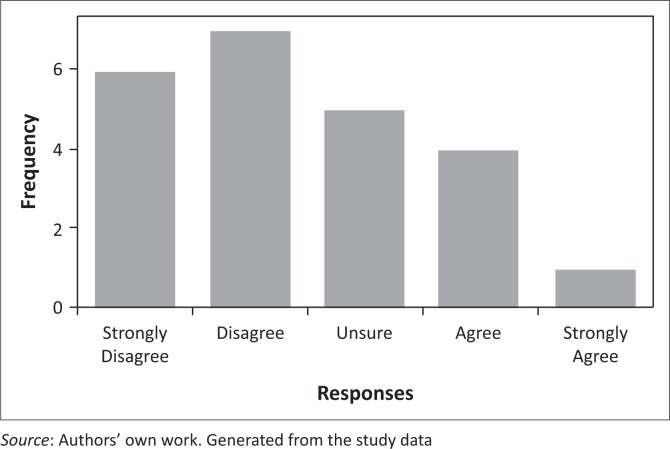
Scaled responses regarding the sufficiency of the funding to support research concerning malaria elimination in SA.

Most researchers (76.9%) participating in this study were of the view that their role in implementing a malaria elimination policy was both advisory and supportive, specifically to produce research evidence on effective interventions to enable successful policy implementation. Of these researchers who viewed their roles as both advisory and supportive, 55% were SAMEC members and 45% were not. At least 56.5% of the participants were somehow unsure about their satisfaction levels regarding their roles in supporting or guiding the implementation of a malaria elimination programme. The remaining 30.4% and 13.0% were somewhat satisfied and very satisfied, respectively. Some participants (61.5%) listed a number of things they disliked about the malaria elimination policy in its current form, including the 2018 target, the lack of focus on curbing the imported infections, insufficient focus on increasing funding, and overreliance on partners to drive elimination initiatives rather than building capacity within the programmes. Respondents had mixed views on whether SA has sufficient number of malaria researchers to guide malaria elimination programmes (Figure 2). However, most of them were adamant that the funding to conduct malaria research in SA is lacking (Figure 3).

### Researchers’ perceptions of facilitators and barriers to malaria elimination in South Africa

**FIGURE 4:**
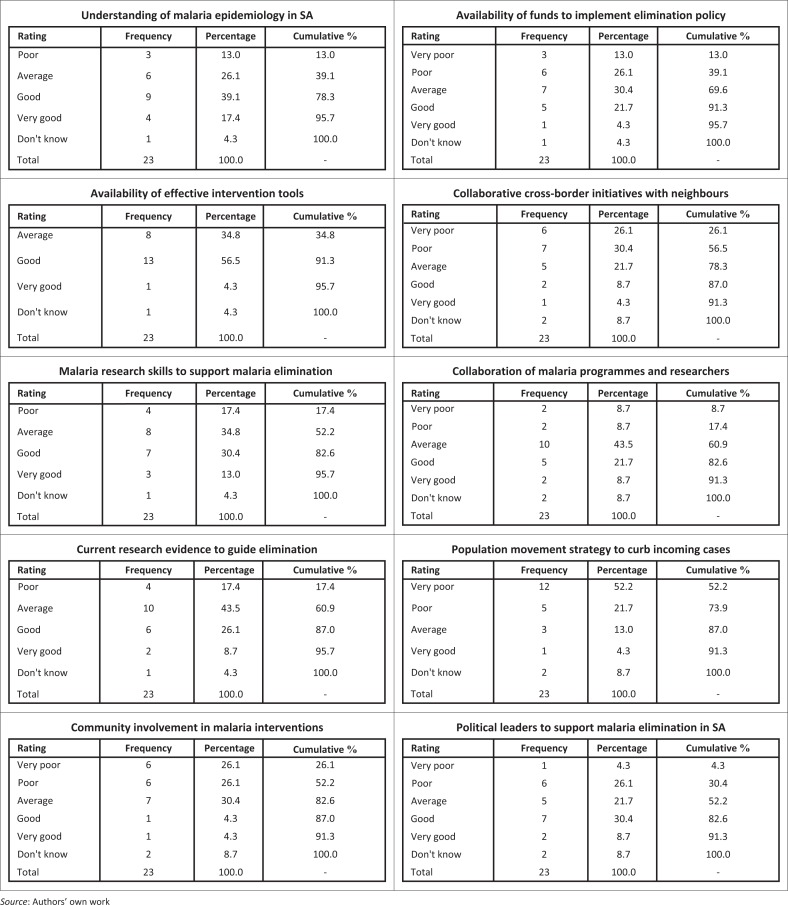
Respondents rating of South Africa with respect to the following key areas.

Participants were asked to rate SA with regards to a range of statements pertaining to malaria elimination. The understanding of malaria epidemiology in SA was given a positive rating, whereby 56.5% of the respondents rated it as either good (39.1%) or very good (17.4%). Participants were also positive about the availability of effective malaria intervention tools in SA. The rating for the availability of malaria research skills to conduct studies to support malaria elimination was neither decisively negative nor positive (Figure 4). Almost two-fifths (38.5%) of the participants were from the universities, whereas 61.5% were mainly from the research institutions and other organisations interested in malaria research. Most participants (60.9%) rated the availability of the contemporary research evidence to guide malaria elimination either as poor (17.4%) or average (43.5%). Similarly, most participants rated the involvement of community in malaria interventions/activities as very poor (26.1%), poor (26.1%), and average (30.4%), respectively. Community involvement, first and foremost, entailed community buy-in to the concept of malaria elimination and subsequently supporting all interventions implemented by malaria programmes, including the adoption of an early treatment-seeking behaviour when malaria infection is suspected.

Availability of funds to implement malaria elimination was poorly rated, so was the collaborative cross-border malaria interventions with endemic neighbouring countries (Figure 4). Collaboration between malaria programmes and research institutions was considered average by 43.5% (Figure 4). Most (73.9%) participants considered SA to lack the strategy for population movement to curb the importation of malaria cases. Notably, the support for malaria elimination by the political leadership did not receive a low rating because 30.4% rated it as good (Figure 4).

**FIGURE 5:**
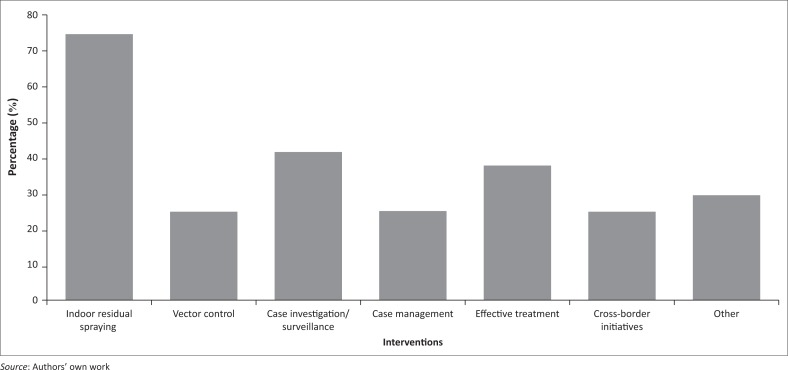
Interventions believed to have substantially reduced malaria cases (*n* = 24) (multiple responses were allowed).

The majority of participants (75%) overwhelmingly identified indoor residual spraying (IRS) as an intervention that had the biggest impact in reducing malaria, as was case investigation/surveillance (41.7%), and effective treatment (37.5%) especially using artemisinin combination therapies (Figure 5). Other (29.2%) identified interventions were better advocacy and awareness about malaria, control of mosquito breeding sites, better community participation, use of community-based spray teams, use of effective insecticides, geographic information system based malaria information system, and the political will.

**FIGURE 6:**
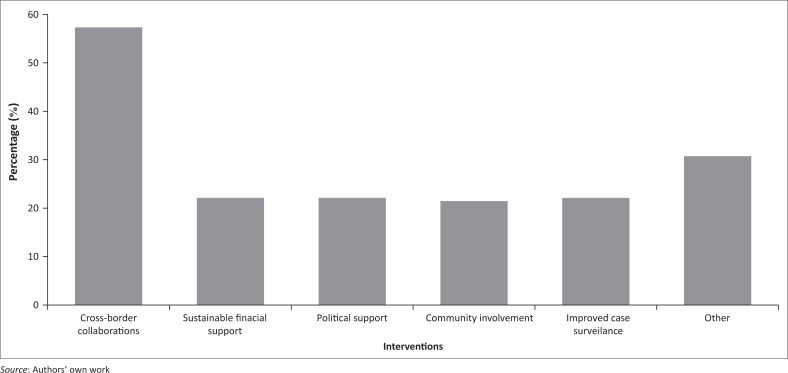
Interventions anticipated to facilitate the success of malaria elimination (*n* = 23) (multiple responses were allowed).

Participants were further asked to list the key things which they believed would make the implementation of a malaria elimination policy a success. Key amongst them was the need for strong cross-border collaborations with the neighbouring malaria endemic countries (56.6%), with 21.7% mentioning sustainable financial support, political support, community involvement, and improved case surveillance (Figure 6). Other (30.4%) suggestions anticipated to make the implementation of a malaria elimination policy succeed included the introduction of presumptive treatment in households of passive cases, regular monitoring of insecticide and drug resistance, staff capacitation, and investing in developing new intervention strategies.

**FIGURE 7:**
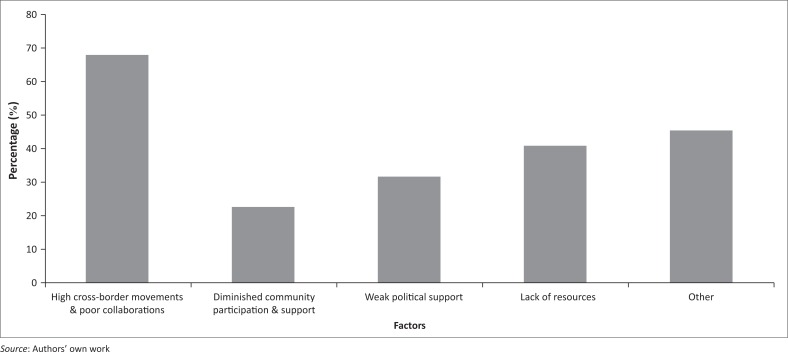
Factors likely to hinder the success of malaria elimination programme (*n* = 22) (multiple responses were allowed).

The commonly listed potential barriers to the success of malaria elimination were high cross-border movements, poor cross-border collaborations between SA and malaria endemic neighbouring countries (68.2%), and the lack of financial and human resources (40.9%). Diminished community involvement and support of malaria interventions (22.7%) and weak political support (31.8%) were also listed as additional barriers (Figure 7). While the supposedly weak political support was surprising, given the high level initiatives, such as the Elimination 8 and African Leaders Malaria Alliance, respondents appeared to judge the strength of political support using inadequacy of funds that political heads deploy to malaria programme activities, other than the number of pacts signed. Other (45.5%) perceived barriers were inability of major malaria control players to listen to those of different opinions, lack of the policy awareness by the affected communities, limited intervention tools, lack of research on how to stop the use of IRS as the cases decrease, imperfect implementation of the IRS programme, poor surveillance, shortage of entomologists, lack of marketing and advocacy strategy, lack of collaboration between research institutions and the Department of Health, and the potential insecticide and drug resistance.

Less than half (46.2%) of the participants believed that malaria elimination differs from the normal control interventions, pointing to the fact that elimination puts greater emphasis on some key areas. These areas include improved information systems and epidemic forecasting, increased focus on active case detection to prevent onward transmission while ensuring proper case classification and spatial epidemiology, increased focus on identifying foci of transmission to allow for targeted implementation of interventions, and increased focus on identifying and treating asymptomatic malaria carriers in an attempt to disrupt the transmission cycle. In a nutshell, all participants were in agreement that elimination does not fundamentally differ from the control, except that the goal post changes in elimination, which has more scaled up interventions with extra specific and tangible targets/outcomes.

## Discussion

In total, 26 (44.1%) of eligible and traceable researchers participated in the study. Although this response rate was disappointing, it was not peculiar to other email surveys,^14^ hence the study conducted amongst the professors of dermatology through an emailed questionnaire in the United States had a comparable (48%) response rate.^15^ Evidence suggests that all web-based surveys generally have lower response rates.^16,17^ Review studies have shown that web-based or emailed questionnaires have varying response rates of between 11% and 70%.^17^ In a study conducted amongst the researchers in 10 countries focusing on bridging the gaps between research, policy, and practice, response rates at the level of single countries ranged from 30% to 100%.^18^ In a study comparing email, fax, and postal surveys of paediatricians, overall response rates were 47%, 57%, and 55%, respectively.^19^

The articles cited above deal more with the factors affecting the response rate than they do with how poor response rate affected the study outcomes.^14,16^ Fan and Yan^16^ identified the research topic, length of the survey, wording, complexity, and biased questions as the key factors affecting the response rates. In this study, 90% of the refusals were actually from the universities, resulting in skewed response rate towards the research institutions and other organisations interested in malaria research (61.5%) compared to the universities (38.5%). Therefore, the university-based researchers’ perspectives on the implementation of malaria elimination policy were not adequately explored. Again, poor response rate limited the investigators from engaging on a robust statistical analysis which could have led to meaningful conclusive findings.

Most participants in this study were of the view that malaria elimination policy has neither been properly adapted to SA’s malaria operational setting nor sufficiently disseminated to all relevant healthcare workers, hence the involvement by all relevant role players was considered to be lacking, especially in the malaria affected communities. Contrary to the WHO view that the country targeting malaria elimination should possess sufficient evidence demonstrating that elimination is a realistic goal,^12^ most researchers held a strong view that the 2018 malaria elimination target was not realistic for SA.

There were strong sentiments suggesting that malaria elimination in SA is overly reliant on partners, thus missing the opportunity to build capacity within the malaria programmes. Most participants considered SA’s malaria programmes to lack funding, and the human and skills capacity to implement malaria elimination. The literature has already suggested that implementing a malaria elimination programme would inevitably need to address constraints relating to financial, operational, and technical considerations.^6,8,20^ There is no suggestion that these constraints have adequately been addressed in SA. Brooke et al.^21^ argued that successful elimination of malaria in SA is dependent on more rather than less resources being invested into malaria control.

Similar to the concerns documented in the literature,^7^ participants in this study were concerned about the lack of new tools to fight malaria. Congruent with El-Moamly’s^6^ findings, which identified vector resistance to insecticides as a potentially serious obstacle to achieving malaria elimination, this study revealed that malaria researchers were concerned about the lack of preparedness with new alternative drugs and insecticides, should resistance to current drugs and insecticides occur. Although malaria researchers participating in this study acknowledged that their role in the implementation of a malaria elimination policy was advisory and supportive in nature, through which they are expected to produce research evidence to guide the elimination programme,^6^ they argued that the current state of malaria research to guide malaria elimination in SA was poor. They attributed this problem to the funding constraints to conduct malaria research in the country.

The study participants considered the importation of malaria cases from the neighbouring malaria endemic countries to be a major barrier to the successful implementation of malaria elimination policy in SA. This phenomenon is neither unique nor new, as Saudi Arabia and Jordan grappled with the same problem, whereby at one point, all the reported cases in these countries were imported.^22,23^ This is a problem, especially when vectors are still present in the country targeting elimination and are in contact with the population, because local transmission is likely to recur.^24^ The study participants were concerned about SA’s weak cross-border controls and collaborations with neighbouring malaria endemic countries. It should be noted that since the collection of data for this study (late 2014 to early 2015), new initiatives, such as the MOSASWA (cross-border collaboration between Mozambique, SA, and Swaziland) have been put in place. Launch of MOSASWA in July 2015 rekindled hope in a situation which was already seen as likely to compromise the gains made towards achieving malaria elimination in the country.

The last strong cross-border collaboration involving SA was the Lubombo Spatial Development Initiative (LSDI) region, a collaborative approach to malaria control between Mozambique, Swaziland, and SA. This collaboration managed to reduce malaria by more than 90% in the targeted areas.^25^ However, this collaboration did not survive beyond the 2012 funding term by the Global Fund to Fight AIDS, Tuberculosis, and Malaria (Global Fund).^25^ Feachem et al.^26^ have acknowledged that establishing and sustaining effective cross-border collaboration is difficult, especially when it comes to financing and managing such funding by donor funders. Accountability becomes particularly difficult to manage and/ or sustain. Hopefully, the newly established MOSASWA will be equally effective in reducing malaria transmission to very low levels.

Diminished community involvement and support for ongoing malaria interventions was considered to be a serious threat to the success of the malaria elimination initiative in SA, as has been the case elsewhere in the world.^27^ The difficulty in obtaining and maintaining community enthusiasm and participation in strategies to eliminate malaria as the disease disappears, has already been documented.^28^ The study conducted in Vanuatu proved that community involvement and support are key contributors to the success of malaria elimination.^28^ Strong and sustainable political and financial support need to be amongst the key pillars of the successful implementation of a malaria elimination programme.

## Conclusion

Although the move to eliminate malaria in SA and other countries is a noble idea, this agenda is surrounded with controversies. According to El-Moamly,^6^ some experts viewed the proposition to eliminate malaria as naïve, whereas others were encouraged by eradication of small pox and believe that it can also be done with malaria. The anti-elimination group fears that if expectations to eliminate malaria are raised and fail again, that this may be a major setback to the malaria control community, in the light of the challenges experienced in the GMEP of 1955–1969. The overarching issue regarding malaria elimination is that it should be evidence-based and express a realistic goal.^20^

Although the move to eliminate malaria is thought to have erupted dichotomous views, namely those who are for the idea and those who are against the idea, in reality there are three viewpoints. These viewpoints are: (a) those who do not believe in malaria elimination; (b) those who believe malaria elimination is worth trying in the future, but this is not the right time; and (c) those who believe malaria can be eliminated and the time to eliminate it is now. Irrespective of the diversity of viewpoints, there is a general agreement that elimination requires: (a) strong cross-border initiatives; (b) deployment of adequate resources; (c) sustainable multistakeholder support and collaboration; (d) good surveillance systems; and (e) availability and use of all effective intervention tools.

Finally, it is worth noting that, although this study has produced some insightful findings, the sample size was too small to warrant any generalisation or to advance authoritative recommendations. The seemingly less sensitive selection criteria was an important limitation of this study. As a result, it would be worthwhile to conduct a similar study with a larger sample size in comparable settings implementing malaria elimination. Acquiring a large sample size would require a more sensitive selection criteria to improve participation rate. One possible way of doing that would be to relax publication criteria used in this study, by ensuring that all authors, irrespective of the position in the authorship list, are eligible for selection. In addition, all postgraduate students whose research thesis involved malaria elimination should be included, irrespective of whether the results have been published or not, because they are anticipated to push towards elimination. This will help increase sample size, while ensuring that the views of the upcoming researchers are heard.

Some innovative thinking in study design would be required to increase the response rate in this population. Some options that come to mind involve internet-based data collection methods, including internet-based data collection methods (such as Survey Monkey), which have produced promising results in other studies.^14^ Another very likely option would be to target international malaria conferences, such as, Multilateral Initiative on Malaria (MIM), as such conferences are known for bringing most malaria researchers and other important stakeholders under one roof. In some instances, initiating communication via personal assistants (PAs) for senior researchers would be worth a try, as PAs often manage their principals’ diaries. A prescheduled telephonically conducted researcher-administered questionnaire is also worth exploring.
